# The Hapten Design, Monoclonal Antibody Preparation, and Immunoassay Development for Rapid Detection of Isofenphos-Methyl

**DOI:** 10.3390/foods15132325

**Published:** 2026-07-01

**Authors:** Yajie Lei, Yunyun Chang, Wenchong Shan, Miao Wang, Yongxin She, A. M. Abd El-Aty, Jing Wang

**Affiliations:** 1 National Nanfan Research Institute (Sanya), Chinese Academy of Agricultural Sciences, Sanya 572024, China; caas_leiyajie@163.com (Y.L.); changyunyun@caas.cn (Y.C.); shanwenchong@caas.cn (W.S.); wm0510@126.com (M.W.); 2 Institute of Quality Standard and Testing Technology for Agro-Products, Chinese Academy of Agricultural Sciences, Beijing 100081, China; 3 Department of Pharmacology, Faculty of Veterinary Medicine, Cairo University, Giza 12211, Egypt; abdelaty44@hotmail.com; 4 Department of Medical Pharmacology, Medical Faculty, Ataturk University, Erzurum 25240, Turkey

**Keywords:** isofenphos-methyl, hapten prediction, monoclonal antibody, indirect competitive enzyme-linked immunosorbent assay

## Abstract

Isofenphos-methyl (IFP), a highly toxic and persistent organophosphate pesticide (OP), is widely used for soil pest control in crops but poses severe risks to ecological safety and human health because of its environmental accumulation and bioaccumulation. Herein, a sensitive and specific indirect competitive enzyme-linked immunosorbent assay (ic-ELISA) was developed for rapid IFP detection in vegetables. A novel IFP hapten was rationally designed and synthesized via computer-aided molecular simulation, and its structure was validated by liquid chromatography–tandem mass spectrometry (LC–MS/MS) and nuclear magnetic resonance (NMR). High-specificity anti-IFP monoclonal antibodies (mAbs) with strong anti-matrix interference were prepared for the first time using a matrix effect-enhanced screening strategy. The optimized ic-ELISA showed high sensitivity, with an IC_50_ of 6.087 ng/mL and a detection range of 1.165–30.490 ng/mL, and no cross-reactivity with other common OPs. Spiked recovery experiments in celery and chili pepper matrices yielded recoveries of 81.87–97.95% (RSD < 5.44%), with highly consistent LC–MS/MS results. The method exhibited a weak positive matrix effect in vegetable matrices, eliminating complex pretreatment and enabling rapid onsite detection.

## 1. Introduction

In recent years, the growing global demand for agricultural productivity has driven the extensive application of pesticides in crop cultivation, which has effectively improved yield and quality while also posing potential environmental residue and food safety hazards [1,2]. IFP, a typical highly toxic organophosphorus pesticide (OP), exerts its insecticidal activity by irreversibly inhibiting acetylcholinesterase activity in target organisms, leading to the disruption of neural signal transmission [3]. It has been widely used in the control of soil pests in food and economic crops such as wheat, corn, and sugarcane because of its broad insecticidal spectrum and strong soil permeability [4,5]. However, IFP is highly environmentally persistent, with a long soil half-life, and it is prone to bioaccumulation and biomagnification through the food chain, posing severe threats to ecosystems, nontarget organisms, and human health [6]. Acute exposure to IFP can induce neurotoxic symptoms in humans and animals, whereas chronic low-dose exposure may cause potential damage to the nervous, digestive, and reproductive systems [3,7]. Moreover, IFP residues exceeding the regulatory limit have been frequently detected in agricultural products such as cowpeas, chives, and tea in recent market surveys, which not only endanger consumer health but also exacerbate the risk of trade barriers for agricultural product exports. To address these risks, strict regulatory standards have been formulated globally, with China’s GB 2763-2026 stipulating a maximum residue limit (MRL) of 0.01 mg/kg for IFP in vegetables, underscoring the urgent need for accurate, sensitive, and rapid detection techniques for IFP residue monitoring [8].

Traditional analytical methods for IFP residue detection include mainly gas chromatography (GC) [9], gas chromatography–tandem mass spectrometry (GC–MS/MS) [10], liquid chromatography–tandem mass spectrometry [11], surface-enhanced Raman spectroscopy (SERS) [12], lateral flow biosensors [13], and supercritical fluid chromatography–tandem mass spectrometry (SFC–MS/MS) [4]. These instrumental methods offer high detection sensitivity and qualitative and quantitative accuracy and are regarded as the gold standard for pesticide residue analysis [14]. However, their practical application is severely limited by inherent drawbacks, such as expensive instrumentation, complex and time-consuming sample pretreatment, reliance on professional operators, and inability to achieve onsite rapid screening [13]. Although some emerging detection technologies, such as electrochemical sensors [15] and molecularly imprinted polymer-based methods [16], have been developed for OP detection, the former are susceptible to electrode material performance and environmental factors, leading to poor reproducibility [6], while the latter face challenges such as low recognition specificity and complicated preparation processes, making it difficult to meet the demand for large-scale onsite detection of IFP residues in agricultural production and food safety supervision. Notably, current research on rapid IFP detection is extremely scarce, with only a single report on an aptamer-based lateral flow biosensor [13], and no systematic immunoanalytical methods have been established for IFP, leaving a critical technical gap in onsite rapid screening of this high-risk pesticide.

Immunoanalytical methods, such as enzyme-linked immunosorbent assays (ELISAs) and immunochromatographic assays (ICAs), have become a research hotspot in the field of rapid pesticide residue detection because of their advantages of high sensitivity, strong specificity, simple operation, low cost, and portability [17,18,19]. The core of developing high-performance immunoassays lies in the preparation of highly specific and sensitive mAbs, while the rational design and synthesis of haptens are the prerequisite and key for obtaining high-quality antibodies [20,21,22]. As a small molecular compound with a molecular weight less than 1000 Da, IFP lacks immunogenicity and cannot directly induce the immune system to produce specific antibodies. It is therefore necessary to modify its molecular structure by introducing appropriate spacer arms and active functional groups and then coupling the modified hapten with macromolecular carrier proteins to form a complete antigen [23,24]. Rational hapten design for IFP must adhere to two key principles: retaining the characteristic structural moieties of the parent compound to ensure subsequent specific antibody recognition and introducing spacer arms with suitable length and active groups (e.g., -COOH, -NH_2_) to improve coupling efficiency with carrier proteins and fully expose antigenic epitopes [23,24]. Previous studies on pesticide hapten design have confirmed that factors such as hapten rigidity, spacer arm length, and the position of active group introduction significantly affect antibody performance and subsequent immunoassay sensitivity [21,24], providing important theoretical and experimental references for IFP hapten design.

In this study, computer-aided molecular simulation technology was used to guide the rational design and screening of IFP haptens, with the characteristic structural moieties of IFP retained and appropriate carboxyl-terminated alkyl spacer arms introduced to synthesize haptens with good structural compatibility and immunogenicity. The synthesized haptens were verified and characterized using high-performance liquid chromatography (HPLC), high-resolution mass spectrometry (HRMS), and nuclear magnetic resonance spectroscopy. The qualified haptens were then coupled with bovine thyroglobulin (BTG) and bovine serum albumin (BSA) via the active ester method to prepare immunogens and coating antigens, with the coupling ratio and successful preparation of complete antigens verified by a NanoDrop Microvolume Spectrophotometer. BALB/c mice were immunized with the optimized immunogen, and hybridoma technology was used to prepare high-specificity and high-sensitivity anti-IFP mAbs. Conventional instrumental analysis methods and immunoassays are often susceptible to matrix interference from complex agricultural samples, which may lead to false positive results or inaccurate quantitative detection. To address this common technical challenge, a matrix effect-enhanced screening strategy was adopted during hybridoma selection, using an actual vegetable sample extraction solution instead of pure buffer to simulate the real matrix environment, which enabled us to obtain antibodies with inherent strong anti-matrix interference ability. The antibody titer, specificity, and affinity were evaluated by indirect ELISA. On this basis, an ic-ELISA method for the rapid detection of IFP was established and systematically optimized, including the determination of the optimal coating antigen concentration, antibody dilution ratio, and reaction conditions. Finally, the practical applicability and accuracy of the established ic-ELISA method were validated by detecting IFP residues in actual agricultural products (celery and chili pepper) with sample recovery experiments and parallel comparisons with standard instrumental analysis methods (LC–MS/MS). This study aims to fill the technical gap in IFP immunoanalytical detection, provide a sensitive and rapid onsite detection method for IFP residue monitoring, and lay a solid foundation for the subsequent development of more portable immunoassay platforms (e.g., colloidal gold ICA test strips). These results provide important technical support for agricultural product quality and safety supervision, environmental risk assessment of OPs, and the implementation of strict control measures for banned and restricted pesticides.

## 2. Materials and Methods

### 2.1. Reagents and Materials

Isofenphos-methyl, isocarbophos, profenofos, parathion-methyl, triazophos, pyraclofos, fenthion, chlorpyrifos, acephate, phorate, methamidophos, and parathion were secured from TanMo Reference Materials Company Ltd. (Changzhou, China). Bovine serum albumin (BSA), bovine thyroglobulin (BTG), N, N-dimethylformamide (DMF), N-hydroxysuccinimide (NHS), 1-ethyl-3-(3-dimethylaminopropyl) carbodiimide (EDC), Freund’s adjuvant, Freund’s incomplete adjuvant, and HRP-sheep anti-mouse IgG enzyme-labeled secondary antibody were procured from Sigma-Aldrich (Shanghai, China). Cell culture media and related reagents were obtained from Gibco BRL (Paisley, UK). Celery and chili peppers were purchased from local supermarkets. 3, 3′, 5, 5′-Tetramethylbenzidin (TMB) was purchased from Biodragon (Suzhou, China). Phosphate-buffered saline (PBS) (0.01 mol/L, pH 7.4), coating buffer (0.1 mol/L carbonate buffer, pH 9.6), wash buffer PBST (0.02 mol/L PBS), blocking solution (2% skim milk powder), and stop solution (10% H_2_SO_4_) were prepared according to the standard methods.

### 2.2. Hapten Design and Screening

With respect to small molecular compounds, the molecular structure of haptens is crucial for inducing the production of high-performance antibodies [25]. To generate monoclonal antibodies with high specificity and affinity for IFP, the designed IFP haptens were engineered to match the target analyte as closely as possible in terms of molecular size, spatial conformation, and electronic property parameters. In addition, the binding site between the hapten and carrier protein, as well as the type and length of the spacer arm, significantly influence the performance of the generated antibodies [26]. Rational hapten design, achieved through the analysis of spatial conformations and epitope information of both the target molecule and hapten, can effectively increase the success rate of antibody preparation and reduce experimental costs.

For the design of IFP haptens, ORCA 6.0 and Discovery Studio 2019 software were employed to perform molecular dynamics simulations for hapten epitope prediction. Electrostatic surface analysis was conducted to quantitatively determine the molecular surface electrostatic potential (ESP) and average local ionization energy (ALIE), which enabled the identification of the characteristic functional groups in the IFP molecular structure. On this basis, the characteristic structural moieties of IFP were retained and fully exposed; alkyl spacer arms were extended, and carboxyl active groups were introduced to synthesize IFP haptens with physicochemical properties, including three-dimensional structure, charge distribution, and hydrophobicity, that satisfy the requirements for efficient antibody recognition, and the corresponding artificial complete antigens were further prepared [5]. IFP haptens were synthesized via a four-step chemical reaction, and NMR spectroscopy and mass spectrometry (MS) were used to verify the molecular structure of the haptens, the successful introduction of active groups, and the accuracy of their molecular weights.

To prepare complete antigens, the active ester method was adopted to couple carrier proteins to the carboxyl groups of the above-synthesized IFP haptens [27]. The haptens were separately conjugated with BTG and BSA to prepare the immunogen and coating antigen, respectively. NanoDrop spectrophotometry was then used to determine the coupling efficiency and the exact molar coupling ratio of the complete antigens. BALB/c mice aged 7 weeks were immunized with the immunogen conjugated with BTG, and the rationality of the designed hapten molecules was further verified by monitoring the serum antibody titer and inhibition rate.

### 2.3. Synthesis of the Hapten

**Figure 1:**
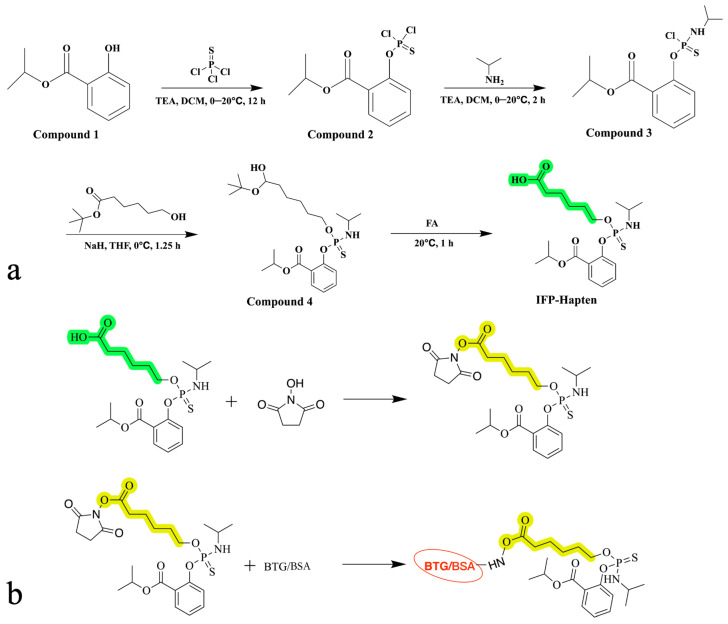
Synthesis pathways of IFP hapten (**a**) and complete antigens (**b**). Green parts: artificially introduced carboxyl alkyl chain, the modified site distinguishing IFP-hapten from parent isofenphos-methyl, serving as protein-coupling site. Yellow parts: activated ester formed from hapten carboxyl and NHS for carrier conjugation. Red circled BTG/BSA: carrier protein covalently bound to hapten to prepare immunogen.

IFP hapten and complete antigens were synthesized through a series of chemical reactions (Figure 1a).

First, the thiophosphoryl chloride intermediate was prepared. Isopropyl salicylate (Compound 1) was reacted with thiophosphoryl chloride in dichloromethane (DCM) using triethylamine (TEA) as the base under a nitrogen atmosphere at −10 °C to 20 °C for 12 h, which directly yielded isopropyl 2-dichlorophosphinothioyloxybenzoate (Compound 2), a dichlorothiophosphoryl-containing intermediate, without further purification.

Second, an amino substitution reaction occurs. A chlorine atom substitution reaction (monochlorine substituted by an isopropylamino group) was conducted with isopropyl 2-dichlorophosphinothioyloxybenzoate (Compound 2) and isopropylamine at 0 °C under the catalysis of TEA. The product was purified by silica gel column chromatography with a gradient elution of 0–10% ethyl acetate/hexane, affording isopropyl 2-[chloro-(isopropylamino) phosphinothioyl] oxybenzoate (Compound 3) as a pale yellow oil.

Third, alkoxy coupling reactions occur. Nucleophilic substitution of isopropyl 2-[chloro-(isopropylamino) phosphinothioyl] oxybenzoate (Compound 3) with 6-(tert-butoxy)-6-oxohexan-1-ol was carried out in tetrahydrofuran (THF) at 0 °C using sodium hydride (NaH) as the base, yielding isopropyl 2-[(6-tert-butoxy-6-oxo-hexoxy)-(isopropylamino) phosphinothioyl] oxybenzoate (Compound 4), a hexanoic acid chain-containing intermediate, as a yellow oil. The reaction mixture was subjected to liquid–liquid extraction followed by silica gel column chromatography purification with a gradient elution of 5–7% ethyl acetate/hexane.

Finally, formic acid deprotection and carboxylic acid formation occur. Isopropyl 2-[(6-tert-butoxy-6-oxo-hexoxy)-(isopropylamino) phosphinothioyl] oxybenzoate (Compound 4) was hydrolyzed in formic acid at room temperature for 1 h to generate the target carboxylic acid. The crude product was sequentially purified by silica gel column chromatography (25–35% ethyl acetate/hexane) and preparative high-performance liquid chromatography (preparative HPLC), ultimately yielding the isofenphos-methyl hapten, 6-(((2-(isopropoxycarbonyl) phenoxy) (isopropylamino) phosphorothioyl) oxy) hexanoic acid.

### 2.4. Preparation of the Immunogen and Coating IFP Antigens

Small-molecule haptens possess antigenicity but lack intrinsic immunogenicity [19], and their immunogenicity can be elicited only by covalent conjugation to macromolecular carriers, typically proteins. BTG is a widely used immunogen carrier owing to its favorable physicochemical stability, abundant free amino groups, low cost, and ready availability [28]. It exhibits excellent solubility across a broad range of pH values and ionic strengths and remains stable in the presence of certain organic solvents, making it an ideal macromolecular scaffold for hapten conjugation. In contrast, compared with other carrier proteins, BSA features relatively weak immunogenicity, which renders it a suitable candidate as a coating antigen carrier for antibody screening and subsequent immunoassay development. In this study, BTG and BSA were selected as the carrier proteins to conjugate with the IFP hapten for the preparation of the immunogen (IFP–hapten–BTG) and coating antigen (IFP–hapten–BSA), respectively. Covalent linkages between the hapten and carrier protein are predominantly achieved via the coupling reaction of reactive functional groups, with carboxyl-amino group conjugation being the most commonly adopted strategy. To prepare immunogenic IFP complete antigens, the carboxyl group of the IFP hapten was activated using the active ester method, followed by conjugation to the free amino groups of the carrier proteins; the reaction schemes for hapten activation and subsequent protein conjugation are illustrated in Figure 1b [29].

Given the presence of a reactive carboxyl group on the IFP hapten that is capable of reacting with the amino groups of carrier proteins, the active ester method was employed for the synthesis of the complete antigens (IFP–hapten–BTG and IFP–hapten–BSA). The optimal molar ratios of hapten to carrier protein were set at 60:1 for the immunogen and 50:1 for the coating antigen. The EDC/NHS-mediated active ester method was used for the conjugation, and the detailed synthetic protocol was as follows: First, the IFP hapten, NHS, and EDC were dissolved in DMF to form Solution A, which was magnetically stirred at room temperature for 4–6 h to achieve full activation of the hapten’s carboxyl group. Separately, 6 mg of BTG was dissolved in 2 mL of carbonate–bicarbonate buffer solution (0.05 mol/L, pH 9.6) to prepare Solution B. Solution A was then slowly added dropwise to Solution B with constant stirring, and the mixture was subjected to dialysis against ultrapure water at room temperature for three days to remove unreacted small molecules, yielding the IFP hapten–BTG immunogen. The synthetic procedure for the IFP hapten–BSA coating antigen was identical to that for the IFP hapten–BTG immunogen.

### 2.5. Production and Performance of Anti-IFP MAbs

Monoclonal antibodies against isofenphos-methyl were prepared following the protocols described in our previous studies [30], which included murine immunization, screening of positive immunized mice, hybridoma cell fusion and subcloning, and ascitic fluid purification. The experimental procedure was consistent with the method established by our research group [31]. All animal experiments were performed in strict accordance with the relevant national laws of China and the ethical guidelines approved by the Experimental Animal Welfare and Animal Ethical Committee of Beijing Kwinbon Biotechnology Co., Ltd. (Approval Code: QBKEGF2024-04, Approval Date: 15 March 2024). Euthanasia of experimental mice was implemented via anesthesia combined with cervical dislocation, a method designed to minimize animal suffering in compliance with the 3Rs principles (Replacement, Reduction, Refinement) of experimental animal ethics.

A modified matrix effect-enhanced screening strategy, which differed from our previous protocols, was adopted in this study to obtain anti-IFP mAbs with superior anti-matrix interference capability. Briefly, to simulate the actual matrix effects of sample detection and ensure that the generated mAbs are applicable for real sample analysis, a sample extraction solution (0.01 M PBS containing 10% methanol, *v/v*) was used to replace the standard 0.01 M PBS buffer in the ic-ELISA during the hybridoma cell screening stage. The isotypes/subtypes of the positive anti-IFP mAbs were identified using a commercially available mouse monoclonal antibody isotyping kit following the manufacturer’s recommended operating instructions.

### 2.6. Indirect Competitive ELISA for the Optimization of Conjugation Protocols

The optimal reaction conditions for the interaction between the IFP antibody and the enzyme-labeled hapten were first determined via the indirect competitive checkerboard titration method to identify the optimal dilution ratios of the coated antibody and the enzyme-labeled hapten. The optimal reaction concentrations were defined as the dilution concentrations of the IFP antibody and enzyme-labeled hapten that yielded an absorbance (OD) value of approximately 1 in the control wells with the maximum inhibition rate. The basic procedures of the indirect competitive checkerboard titration were consistent with those described previously [32], and an IFP inhibition concentration of 10 ng/mL was selected for the titration. To develop an ic-ELISA method with high stability and sensitivity and obtain the optimal reaction conditions, it was necessary to first screen a sample extraction solution with anti-matrix interference capability to determine the most suitable methanol concentration. On the basis of the optimized reaction conditions, the sample diluent was further optimized, including its pH and sodium ion (Na^+^) concentration. Serial concentrations of the IFP standard (0, 0.625, 1.25, 2.5, 5, 10, 20, and 40 ng/mL) were detected under different competitive reaction times, with three replicate wells set for each concentration. The OD values were measured, and a standard curve was plotted using the ratio of the OD values of the inhibition wells to those of the control wells (B/B_0_) and the corresponding standard concentrations.

With the optimal dilution ratios of the coated antibody and enzyme-labeled hapten fixed, the methanol content in the sample diluent used to dissolve the IFP standard and IFP antibody was adjusted to 0%, 5%, 10%, 15%, 20%, and 40%, without altering the other reaction conditions. After the optimal methanol concentration was determined, the pH of the sample diluent was adjusted to 4.5, 5.5, 6.5, 7.5, 8.5, and 9.5 using 0.1 M hydrochloric acid and 0.1 M sodium hydroxide, respectively. Subsequently, the Na^+^ concentration in the sample diluent was further adjusted to 0.016, 0.05, 0.1, 0.15, 0.2, and 0.4 M. Finally, the competitive reaction time was optimized to 15, 30, 45, 60, and 90 min. To construct the standard curve, the IFP standard stock solution was diluted to the aforementioned serial concentrations using the optimized sample diluent. The ic-ELISA standard curve that exhibited an approximate OD value of 1 and the maximum inhibition rate under the optimal conditions was selected, and the half-maximal inhibitory concentration (IC_50_) was calculated accordingly.

The specificity of the prepared IFP antibody was evaluated using the optimized ic-ELISA method. The IFP and IFP structural analog pesticides (isocarbophos, profenofos, parathion-methyl, triazophos, pyraclofos, fenthion, chlorpyrifos, acephate, phorate, methamidophos, and parathion) were prepared. These 11 organophosphorus pesticides were specifically applied to evaluate the cross-reactivity of the anti-IFP monoclonal antibodies, with their gradient concentrations formulated with the optimized sample diluent. Standard curves for all the analytes were plotted separately, and the cross-reactivity (CR) rates were calculated to assess antibody specificity. For all ic-ELISA experiments, each concentration was tested in triplicate wells (*n* = 3) per plate, and all key experiments were independently repeated three times on different days to ensure reproducibility.

### 2.7. Recovery Test and Real Sample Analysis

Negative celery and chili pepper samples confirmed by LC–MS/MS were selected to evaluate the accuracy of the established ic-ELISA method via spiked recovery tests. Our sample pretreatment protocol strictly follows the pretreatment method specified in Section 7.1 of the National Standard of China GB 23200.121–2021 “Determination of 208 pesticides and metabolites residues in foods—QuEChERS–liquid chromatography–tandem mass spectrometry method” [33]. After extraction, we added a nitrogen blow concentration step and reconstituted the residue with our optimized ELISA buffer to better adapt to the detection conditions of the ic-ELISA method. The reconstituted samples were used for both ic-ELISA and LC-MS/MS detection and analysis to ensure the comparability of the results. To ensure representative results, three spiked concentrations (high, medium, and low) within the detection range of ic-ELISA were chosen as 20 ng/mL, 10 ng/mL, and 5 ng/mL, respectively, and corresponding standard curves were constructed. The accuracy of the method was assessed in terms of recovery rates and relative standard deviations (RSDs). In addition, authentic celery and chili pepper samples were collected, detected by ic-ELISA, and further verified using LC–MS/MS. For spiked recovery experiments and real sample analysis, each spiked concentration was prepared in three independent parallel samples (*n* = 3), and each sample extract was measured in duplicate by both ic-ELISA and LC-MS/MS.

## 3. Results

### 3.1. Hapten Analysis

The fundamental purpose of hapten design is to elicit a specific immune recognition response in the host organism and generate high-affinity antibodies that can specifically bind to the target analyte molecule [34]. Rational hapten design must adhere to two core principles: First, the designed hapten should mimic the target analyte as closely as possible in terms of molecular structure, physicochemical properties, and electrostatic potential (ESP) to ensure that the immune system can effectively recognize the characteristic structural moieties of the analyte [19]; second, a spacer arm with an appropriate alkyl chain length should be introduced between the hapten and carrier protein, and the arm should be designed to avoid inducing the production of nonspecific “spacer arm antibodies” that do not recognize the target analyte [23].

**Figure 2:**
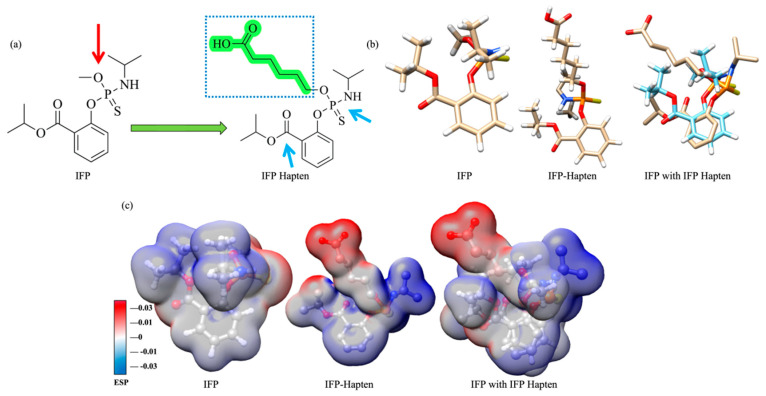
Hapten design and computer-aided analysis results: (**a**) The structure of IFP and IFP hapten. (**b**) The spatial overlap of IFP and IFP hapten. (**c**) ESP distribution of IFP and IFP hapten. Red arrow: the chemical site for structural modification during hapten design. Blue arrows: conserved core skeletons inherited from parent IFP pesticide. Green fragment in dotted box: newly introduced carboxyl alkyl linker, the modified moiety distinguishing IFP‑hapten from native IFP and providing active site for carrier protein conjugation.

IFP, a low-molecular-weight organophosphorus pesticide (MW~331 Da), possesses intrinsic antigenicity but lacks immunogenicity, necessitating structural modification and hapten design to generate specific anti-IFP antibodies. The core structural features of IFP include a phosphorothioamide group, a phosphonyl moiety, and an isopropoxycarbonyl group (Figure 2a), which represent the unique characteristic moieties critical for specific immune recognition. In this study, to minimize structural alterations to the parent IFP molecule while retaining all its key characteristic groups, a carboxyl reactive group (-COOH) was introduced at the P-O-C site of the IFP molecular structure, and an extended alkyl spacer arm was attached to this site for subsequent carrier protein conjugation.

To screen for the optimal IFP hapten, computer-aided molecular simulation technology was employed to conduct a comprehensive conformational and electrostatic potential analysis of the parent IFP molecule and the designed hapten. As shown in Figure 2b, when the IFP molecule was used as the template, the three-dimensional (3D) conformations of IFP and the designed hapten were optimized to their minimum energy states, and the superposition analysis revealed a high degree of structural overlap between the IFP hapten and the parent IFP molecule. Furthermore, the surface ESP distribution profiles of IFP and the hapten (Figure 2c) exhibited consistent overall electrostatic characteristics: the phenyl ring moiety in both structures presented a negative ESP region, whereas the phosphate ester group exhibited a positive ESP region. This structural and electrostatic similarity ensures that the designed hapten can effectively mimic the parent IFP molecule and elicit the production of specific antibodies targeting the characteristic structural moieties of IFP.

For covalent conjugation with carrier proteins (e.g., BTG and BSA), the carboxyl group at the terminus of the hapten’s spacer arm was activated via the EDC/NHS active ester method—a widely used strategy for hapten–carrier protein coupling. This design strategy not only preserves the complete exposure of IFP’s core characteristic groups to the host immune system but also minimizes steric hindrance during antibody recognition and hapten–carrier conjugation, laying a solid foundation for the subsequent generation of high-specificity and high-affinity anti-IFP monoclonal antibodies.

### 3.2. Characterization of the Hapten and Antigen

**Figure 3:**
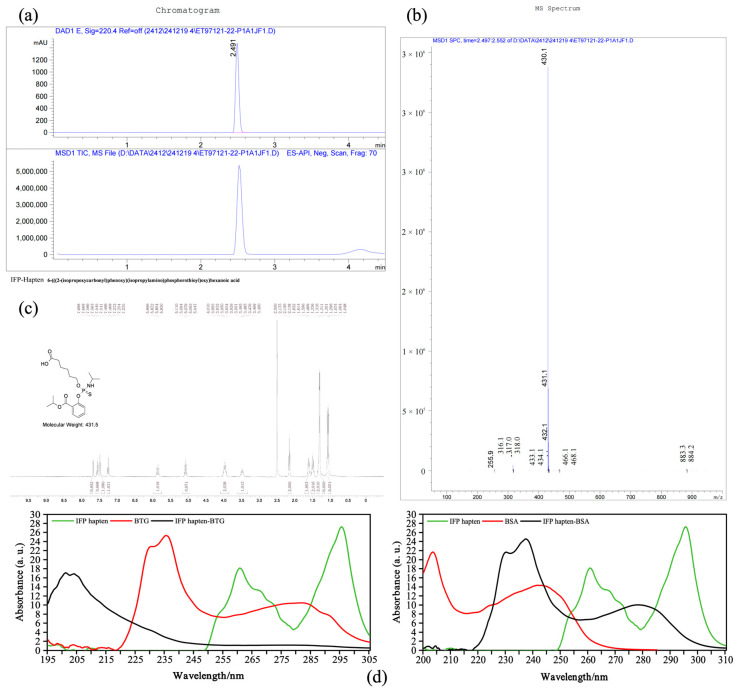
Characterization of the hapten and the complete antigen: (**a**) LC characterization of IFP hapten. (**b**) MS characterization of IFP hapten. (**c**) 1H NMR characterization of IFP hapten. (**d**) The UV-vis results of the complete IFP hapten and the complete antigens.

The synthesis of the IFP hapten was characterized and verified using LC–MS/MS and nuclear magnetic resonance spectroscopy (^1^H NMR and ^31^P NMR) (Figure 3). The theoretical molecular weight of the IFP hapten was 431.5 Da; in negative electrospray ionization mode (ESI-) (Figure 3b), the target hapten exhibited a characteristic retention time of 2.491 min in the LC analysis (Figure 3a), which is consistent with the expected chromatographic behavior.

The detailed ^1^H NMR and ^31^P NMR spectral data (Figure 3c) were as follows: ^1^H NMR (400 MHz, DMSO-d_6_) δ = 7.68 (d, J = 7.6 Hz, 1H), 7.61–7.53 (m, 1H), 7.25 (t, J = 7.5 Hz, 1H), 5.86 (dd, J = 9.6, 16.6 Hz, 1H), 5.08 (quin, J = 6.3 Hz, 1H), 4.10–3.86 (m, 2H), 3.48 (tt, J = 6.4, 10.1 Hz, 1H), 2.16 (t, J = 7.3 Hz, 2H), 1.66–1.57 (m, 2H), 1.50 (quin, J = 7.5 Hz, 2H), 1.38–1.32 (m, 2H), 1.31 (dd, J = 2.1, 6.2 Hz, 6H), 1.07 (dd, J = 6.5, 10.3 Hz, 6H); ^31^P NMR (400 MHz, DMSO-d_6_) δ = 66.56 (s, 1P). The obtained spectral data were in full agreement with the designed chemical structure of the IFP hapten, confirming the successful synthesis of the target hapten.

The successful coupling of the IFP hapten with carrier proteins to form complete antigens (IFP–hapten–BTG and IFP–hapten–BSA) was validated via NanoDrop (Figure 3d). In the UV absorption range of 200–310 nm, the maximum absorption peaks of the carrier proteins (BTG and BSA) occurred at 238 nm and 204 nm, respectively, whereas the maximum absorption peak of the IFP hapten occurred at 295 nm. In contrast, the prepared complete antigens (IFP–hapten–BTG and IFP–hapten–BSA) displayed characteristic maximum absorption peaks at 237 nm and 239 nm, respectively. A distinct redshift or blueshift in the maximum UV absorption peaks was observed for all complete antigens compared with the corresponding free carrier proteins and the IFP hapten, which is a typical spectral signature of the covalent conjugation between small-molecule haptens and macromolecular proteins. These results thus confirmed the successful synthesis of the IFP–hapten–BTG immunogen and IFP–hapten–BSA coating antigen.

### 3.3. Identification of the Performance of Anti-IFP MAbs

**Figure 4:**
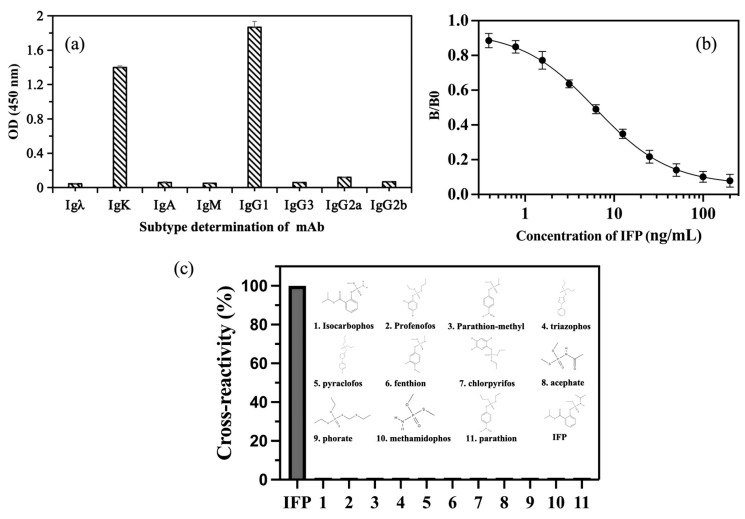
Performance analysis of anti-IFP mAbs: (**a**) Subtype analysis of anti-IFP mAbs. (**b**) ic-ELISA inhibition curve analysis of anti-IFP mAbs. (**c**) Specificity measurement of anti-IFP mAbs.

The preparation of high-performance mAbs plays a crucial role in the development of subsequent immunoassays [35]. Therefore, a sample extraction solution was used instead of ELISA buffer to simulate matrix effects, and the best-performing cell lines (with high titer and high inhibition) were screened to prepare high-performance mAbs with good resistance to matrix interference. Although the cell line 9C5D5C10 showed the best inhibition rate, its low titer was not conducive to the establishment of an immunoassay, whereas the cell line 6D2C8E3 exhibited excellent titer and inhibition rates in the sample extraction solution and was therefore used for the production of the anti-IFP mAbs. Subtype, affinity, sensitivity, and specificity are key indicators for assessing the performance of mAbs [36,37]. Owing to differences in the sequence, number, hinge structure, and antibody combination valency in the constant region of heavy chains, antibodies are classified into different subtypes. Among them, antibodies in mammalian serum are mainly IgG, accounting for approximately 80% of the total amount of immunoglobulins, while IgM and IgA each account for approximately 10% [38]. On the basis of the differences in antibody light chains, they can be divided into kappa and lambda, whose protein sequences differ. The determination of antibody subtypes can further aid in understanding the structure and function of antibodies. As shown in Figure 4a, the heavy chain and light chain were IgG1 and kappa, respectively, with high purity. The affinity of mAbs refers to the noncovalent forces between antibodies and antigenic determinants or antigenic epitopes, including attractions between amino acids, hydrogen bonds, and hydrophobic forces [39]. As important indicators for the evaluation of antibody quality, high-affinity mAbs are beneficial for the development of immunochromatography. As shown in Figure 4b, the anti-IFP mAb sensitivity is reflected by the standard curve, y = 0.061 + 0.8725/(1 + [x/6.087]^−1.063^), which has an IC_50_ of 6.087 ng/mL, indicating high sensitivity. The detection range of the ic-ELISA for IFP was 1.165–30.490 ng/mL (IC_20_–IC_80_). Highly specific antibodies are beneficial for improving the accuracy of the established method and avoiding false positives. As shown in Figure 4c, the anti-IFP mAbs had no cross-reactivity with other organophosphorus pesticides and therefore showed high specificity. Therefore, anti-IFP mAbs have a high recognition ability for IFP, exhibit high specificity and affinity, and can be used for the establishment of subsequent ic-ELISA.

### 3.4. Optimization of the ic-ELISA

The performance of ic-ELISA, such as sensitivity and stability, is influenced by the antigen concentration, antibody concentration, properties of the solution, and competitive reaction time [40,41]. On the basis of the optimal coating antigen and antibody concentration determined by the indirect competitive checkerboard titration method (OD value ≈ 1 with the maximum inhibition rate), the ic-ELISA reaction conditions were systematically optimized by investigating the effects of the methanol concentration, pH value, Na^+^ concentration (ionic strength), and competitive reaction time on the performance of the assay. The optimization was evaluated by key parameters, including the half-maximal inhibitory concentration (IC_50_), IC_20_, IC_80_, and correlation coefficient (R^2^) of the standard curve, where a lower IC_50_ indicated higher assay sensitivity, and a higher R^2^ reflected better goodness of fit of the standard curve. All optimization experiments were performed with a series of IFP standard concentrations (0, 0.625, 1.25, 2.5, 5, 10, 20, and 40 ng/mL), and the standard curves were plotted using the B/B_0_ ratio and corresponding IFP concentrations. Table 1 shows the optimal coating antigen concentration and antibody dilution ratio, with the coating antigen diluted 1000-fold and the antibody diluted 4000-fold as the optimal reaction conditions. Following the confirmation of these optimal concentrations, subsequent optimizations of the methanol concentration, pH value, Na^+^ concentration, and competitive reaction time for the ic-ELISA were conducted sequentially.

#### 3.4.1. Optimization of the Methanol Concentration

**Figure 5:**
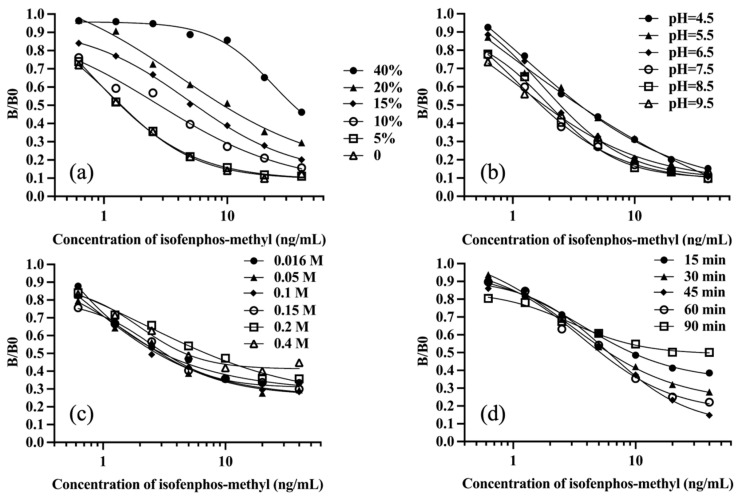
Calibration curves of ic-ELISA against IFP, obtained under different methanol concentrations (**a**), pH conditions (**b**), Na^+^ concentrations (**c**), and reaction times (**d**).

Methanol, a common organic solvent in pesticide sample extraction, can mitigate matrix interference but may also affect the specific binding of antigens and antibodies by altering their hydrophobic interactions and conformational stability [42]. Low concentrations of methanol can reduce non-specific binding between antigens and antibodies and improve assay specificity, while high concentrations may denature antibodies or disrupt the antigen–antibody binding interface, leading to decreased binding affinity and sensitivity. The effects of methanol concentration (0%, 5%, 10%, 15%, 20%, and 40%) on ic-ELISA performance were investigated, and the corresponding IC_50_, IC_20_, IC_80_, and R^2^ values were determined (Figure 5a; Table 2). The results revealed a significant dose-dependent effect of methanol on assay sensitivity: the IC_50_ value decreased sharply with increasing methanol concentration from 0% to 5%, reaching a minimum value of 0.82 ng/mL at 5% methanol, which was accompanied by the lowest IC_20_ (0.31 ng/mL) and a high R^2^, indicating the highest sensitivity and good goodness of fit of the assay at this concentration. When the methanol concentration exceeded 5%, the IC_50_ increased significantly (15%: 4.88 ng/mL; 20%: 4.10 ng/mL; 40%: 23.45 ng/mL), and the IC_20_ and IC_80_ also increased drastically, indicating that the sensitivity of the assay decreased rapidly. This phenomenon suggested that an appropriately low concentration of methanol (5%) could reduce the nonspecific binding between the antigen and antibody, increase the specificity of immune recognition, and thus improve assay sensitivity, whereas high methanol concentrations (>5%) might denature the antibody or destroy the antigen–antibody binding interface, leading to a significant decrease in binding affinity. Therefore, 5% methanol was selected as the optimal concentration for the subsequent ic-ELISA experiments.

#### 3.4.2. Optimization of the pH

The pH of the sample buffer is a critical factor governing the charge distribution, conformational stability, and electrostatic interactions of antigen and antibody molecules and directly affects the sensitivity and reliability of the ic-ELISA [43]. The effect of pH on ic-ELISA performance was systematically evaluated across a range of 4.5 to 9.5, with key performance parameters, including the IC_50_, IC_20_, and IC_80_, and the standard curve correlation coefficient (R^2^), summarized in Figure 5b and Table 3.

As shown in Table 3, the assay sensitivity exhibited a distinct bimodal trend with increasing pH. Under weakly acidic conditions (pH 5.5), the assay achieved the lowest IC_50_ value of 1.05 ng/mL, accompanied by an extremely low IC_20_ of 0.15 ng/mL and a high R^2^ of 0.99, indicating exceptional sensitivity and good goodness of fit. The IC_50_ values at pH 4.5, 7.5 and 9.5 were relatively close (1.26, 1.15 and 1.15 ng/mL, respectively), indicating moderate sensitivity, while the IC_50_ increased significantly to 1.76 ng/mL and 1.95 ng/mL at pH 6.5 and 8.5, respectively, with a sharp increase in the IC_20_ (0.97 ng/mL and 0.99 ng/mL, respectively), confirming a notable decrease in the sensitivity of the assay under these conditions. The optimum performance at pH 5.5 is likely attributable to the isoelectric point (pI) of the anti-IFP monoclonal antibody, as antibody–antigen binding interactions are typically most favorable near the antibody’s pI. Extreme pH values (strong acidity at pH 4.5 and strong alkalinity at pH 7.5–9.5) induce irreversible conformational changes in the antibody, disrupt the antigenic epitope recognition interface, and weaken specific binding interactions, thereby reducing the sensitivity of the assay. All tested pH conditions maintained R^2^ values above 0.98, confirming the stability of the standard curve goodness of fit across the evaluated range. On the basis of the comprehensive analysis of sensitivity (IC_50_/IC_20_) and goodness of fit (R^2^), pH 5.5 was selected as the optimal pH for the sample buffer in the subsequent ic-ELISA experiments, as it delivered the highest assay sensitivity and reliable goodness of fit for IFP detection.

#### 3.4.3. Optimization of Na^+^ Concentration

Ionic strength (regulated by the Na^+^ concentration) affects the antigen–antibody reaction by screening the electrostatic repulsion between charged groups on the molecular surface and maintaining the stability of the microenvironment of the immunoassay system [44]. Insufficient ionic strength leads to increased non-specific binding, while excessive ionic strength causes a “salting out” effect that reduces the solubility of immune complexes. The effects of Na^+^ concentration (0.016 M, 0.05 M, 0.1 M, 0.15 M, 0.2 M, and 0.4 M) on ic-ELISA performance were investigated (Figure 5c; Table 4). The results showed that the assay sensitivity was extremely sensitive to low Na^+^ concentrations: at 0.15 M Na^+^, the IC_50_ was as high as 2.47 ng/mL, and the sensitivity was the lowest among all groups, which might be due to the insufficient ionic strength to screen for nonspecific electrostatic repulsion, leading to weak specific binding of the antigen and antibody. With increasing Na^+^ concentration to 0.05 M, the IC_50_ decreased sharply to a minimum of 0.87 ng/mL, and the IC_20_ (0.15 ng/mL) was low, with a high R^2^, reflecting the optimal balance between ionic strength and antigen–antibody binding at this concentration. When the Na^+^ concentration exceeded 0.05 M (0.1 M, 0.15 M, 0.2 M, and 0.4 M), the IC_50_ increased gradually (0.98, 2.47, 2.20, and 2.02 ng/mL, respectively), and the assay sensitivity decreased slightly because excessive ionic strength would cause a “salting out” effect, reduce the solubility of antigen and antibody molecules, and hinder the formation of specific immune complexes. In a comprehensive analysis of sensitivity and goodness of fit, 0.05 M Na^+^ was selected as the optimal ionic strength for the ic-ELISA method.

#### 3.4.4. Optimization of Competitive Reaction Time

The competitive reaction time directly affects the efficiency and practical applicability of the immunoassay: a short reaction time may lead to incomplete antigen–antibody competition, whereas an overlong time will reduce the detection efficiency and is not conducive to rapid onsite detection. On the basis of the optimized conditions (5% methanol, pH 5.5, and 0.05 M Na^+^), the effects of competitive reaction times (15 min, 30 min, 45 min, 60 min, and 90 min) on ic-ELISA performance were investigated (Figure 5d; Table 5). The results showed that the sensitivity of the assay improved with increasing reaction time within a certain range and then tended to be stable: at 15 min, the IC_50_ was 3.59 ng/mL, which was relatively high, indicating that the competitive reaction was incomplete because the reaction time was insufficient; when the reaction time was extended to 30 min, the IC_50_ decreased significantly to 2.90 ng/mL, and the IC_20_ (1.09 ng/mL) was the lowest among all the groups, with a high R^2^, reflecting that the antigen–antibody competitive reaction essentially reached equilibrium at 30 min and that the sensitivity of the assay was the highest. With further extension of the reaction time (45 min, 60 min, and 90 min), the IC_50_ values increased slightly (5.95, 3.69, and 2.97 ng/mL, respectively), and the sensitivity did not improve but slightly decreased, while the goodness of fit of the standard curve remained stable. Considering both the detection sensitivity and the practical detection efficiency of the immunoassay, 30 min was determined as the optimal competitive reaction time for the ic-ELISA method.

Through the systematic optimization of four key reaction parameters, the optimal conditions for the ic-ELISA method for IFP detection were determined as follows: sample diluent containing 5% methanol, a pH of 5.5, 0.05 M Na^+^, and a competitive reaction time of 30 min. Under these optimal conditions, the ic-ELISA exhibited the lowest IC_50_ value, the strongest antigen–antibody-specific binding ability, good goodness of fit of the standard curve, and high detection efficiency, laying a solid foundation for the subsequent detection of IFP residues in actual samples and the evaluation of the practical applicability of the assay.

### 3.5. Matrix Effect Evaluation via Standard Curve Comparison

**Figure 6:**
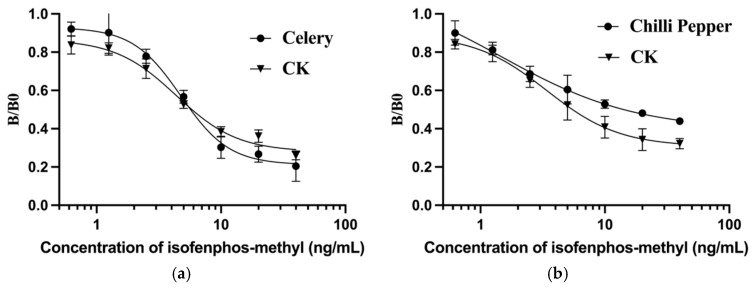
Solvent and matrix-matched ic-ELISA celery (**a**) and chili pepper (**b**) calibration curve of IFP.

Matrix effects are critical factors affecting the accuracy of immunoassays for real sample detection, as endogenous substances in agricultural matrices may cause nonspecific binding or interfere with antigen–antibody-specific recognition [45]. To quantify and mitigate matrix effects, solvent standard curves (no matrix) and matrix-matched standard curves (blank celery/chili pepper extract) were constructed for ic-ELISA, with their key fitting parameters summarized and the curves plotted in Figure 6.

For celery, the solvent standard curve and matrix-matched standard curve both exhibited good goodness of fit, with R^2^ values of 0.9733 and 0.9640, respectively, indicating that the impact of the celery matrix on the linear response of ic-ELISA was negligible. The IC_50_ values of the solvent and celery matrix curves were 4.763 ng/mL and 4.369 ng/mL, respectively—showing a slight decrease in the IC_50_ for the matrix-matched curve, which suggested a weak positive matrix effect (mild enhancement of antigen–antibody binding) in the celery extract.

The chili pepper matrix also showed minimal interference with ic-ELISA, with the solvent and matrix-matched curves achieving comparable goodness of fit (R^2^ = 0.9561 vs. 0.9541). The IC_50_ of the chili pepper matrix curve (1.465 ng/mL) was significantly lower than that of the solvent curve (3.407 ng/mL), indicating a more pronounced positive matrix effect in chili pepper extract, which further improved the sensitivity of the assay for IFP detection in this matrix.

Collectively, the high consistency of goodness of fit, IC_50_, and plateau parameters between the solvent and matrix-matched standard curves for both celery and chili pepper demonstrated that the optimized ic-ELISA method has a strong anti-matrix interference capability. The positive matrix effects observed in both matrices even improved the sensitivity of the assay, which is a rare and favorable characteristic for immunoassays, eliminating the need for complex matrix pretreatment or signal correction in actual sample detection.

### 3.6. Spiked Recovery Analysis of ic-ELISA in Celery and Chili Pepper

To verify the accuracy and reliability of the optimized ic-ELISA for IFP residue detection, blank celery and chili pepper samples were spiked with IFP at three concentrations (5, 10, and 20 ng/mL), and the spiked samples were analyzed by both ic-ELISA and LC–MS/MS (Table 6). The ic-ELISA exhibited satisfactory recovery rates of 81.87–94.91% in celery and 86.18–97.95% in chili pepper, with all relative standard deviation (RSD) values below 5.44%. For LC–MS/MS, the recovery rates ranged from 88.70–94.91% (celery) to 92.63–97.15% (chili pepper), with an RSD ≤ 4.46%. The quantitative results and recovery trends of ic-ELISA were highly consistent with those of LC–MS/MS (the gold standard for pesticide detection), with no significant difference between the two methods. These results confirmed that the ic-ELISA in this study was developed with a matrix effect-enhanced antibody screening strategy and showed strong anti-matrix interference capability in vegetable matrices (celery and chili pepper) without complex matrix correction. It features simple operation, short detection time (30 min competitive reaction), good accuracy, precision, and anti-matrix interference capability, and it is suitable for high-throughput onsite rapid screening of IFP residues in agricultural products at grassroots supervision stations and production bases.

## 4. Conclusions

The core scientific contribution of this study is to fill the long-standing technical gap in the immunoanalytical detection of isofenphos-methyl. We first established a systematic and reliable ic-ELISA method for IFP detection. This achievement is based on two key technical innovations: the computer-aided rational hapten design that retains all characteristic structural moieties of IFP, and the matrix effect-enhanced screening strategy that endows antibodies with inherent anti-matrix interference ability. On the basis of these mAbs, a sensitive and specific ic-ELISA method was established and optimized for IFP detection, with optimal reaction conditions of 5% methanol, pH 5.5, 0.05 M Na^+^, and a 30 min competitive reaction time. The developed ic-ELISA showed high specificity (no cross-reactivity with other OPs) and good anti-matrix interference performance in celery and chili pepper, with satisfactory recovery and precision consistent with the gold standard LC–MS/MS. The method features simple operation, short detection time, and no need for complex sample pretreatment, enabling efficient onsite rapid screening of IFP residues in vegetable samples. It provides a practical technical tool for agricultural product quality safety supervision and environmental risk monitoring of banned/restricted Ops and lays a solid foundation for the development of more portable, rapid IFP detection platforms (e.g., immunochromatographic test strips).

## Figures and Tables

**Table 1:** The titer and inhibition rate of monoclonal antibody 6D2C8E3.

The Concentration of Coated Antigen Diluted	500			1000			2000			5000		
The Concentration of Antibody Diluted	C	I	IR	C	I	IR	C	I	IR	C	I	IR
500	3.550	2.638	0.257	2.144	1.406	0.344	1.193	0.787	0.341	0.684	0.437	0.361
1000	3.245	1.936	0.404	1.750	0.912	0.479	0.918	0.479	0.478	0.488	0.364	0.254
2000	2.626	1.292	0.508	1.529	0.587	0.616	0.774	0.333	0.570	0.363	0.246	0.323
4000	1.857	0.816	0.561	1.227	0.401	0.673	0.540	0.231	0.573	0.290	0.228	0.212
8000	1.313	0.493	0.625	0.729	0.277	0.620	0.396	0.148	0.626	0.217	0.182	0.161
16,000	1.041	0.343	0.671	0.493	0.196	0.601	0.548	0.136	0.752	0.192	0.203	−0.054

Note: C represents the control wells in the microplate, I represents the inhibition wells in the microplate, IR represents the inhibition rate, the coating antigen concentration and antibody concentration are both 1 mg/mL, and the IFP pesticide inhibition concentration is 10 ng/mL.

**Table 2:** The influence of sample buffer methanol concentration on ic-ELISA performance.

Methanol Concentration	40%	20%	15%	10%	5%	0%
IC_50_ (ng/mL)	23.45	4.10	4.88	2.73	0.82	1.20
IC_20_ (ng/mL)	8.92	1.35	1.87	0.85	0.31	0.45
IC_80_ (ng/mL)	61.05	12.47	12.68	8.81	2.13	3.13
Standard curve correlation coefficient R^2^	0.97	0.95	0.96	0.94	0.99	0.99

**Table 3:** The influence of sample buffer pH on ic-ELISA performance.

pH Value	4.5	5.5	6.5	7.5	8.5	9.5
IC_50_ (ng/mL)	1.26	1.05	1.76	1.15	1.95	1.15
IC_20_ (ng/mL)	0.16	0.15	0.97	0.48	0.99	0.26
IC_80_ (ng/mL)	10.07	6.93	3.12	2.68	3.87	4.98
Standard curve correlation coefficient R^2^	0.98	0.99	0.99	0.98	0.99	0.99

**Table 4:** The influence of Na^+^ in sample buffer on ic-ELISA performance.

NaCl Concentration	0.016 M	0.05 M	0.1 M	0.15 M	0.2 M	0.4 M
IC_50_ (ng/mL)	1.66	0.87	0.98	2.47	2.20	2.02
IC_20_ (ng/mL)	0.58	0.15	0.30	0.90	0.52	0.79
IC_80_ (ng/mL)	4.73	0.26	2.05	6.69	9.17	5.21
Standard curve correlation coefficient R^2^	0.98	0.98	0.99	0.99	0.98	0.98

**Table 5:** The influence of reaction time on ic-ELISA performance.

Reaction Time	15 min	30 min	45 min	60 min	90 min
IC_50_ (ng/mL)	3.59	2.90	5.95	3.69	2.97
IC_20_ (ng/mL)	1.318	1.09	2.26	1.38	0.88
IC_80_ (ng/mL)	9.83	7.62	15.44	9.72	9.98
Standard curve correlation coefficient R^2^	0.96	0.96	0.97	0.97	0.95

**Table 6:** Comparison between ic-ELISA and LC-MS/MS analysis for IFP recovery in celery and chili pepper.

Sample	Spiked Concentration (ng/mL)	ic-ELISA			LC-MS/MS		
		Measured Concentration (ng/mL)	Recovery (%)	RSD (%)	Measured Concentration (ng/mL)	Recovery (%)	RSD (%)
Celery	20	18.65 ± 0.81	88.86	4.32	19.46 ± 0.46	94.91	2.37
	10	9.08 ± 0.24	82.00	2.70	9.63 ± 0.43	91.48	4.46
	5	4.97 ± 0.15	81.87	3.10	4.92 ± 0.12	88.7	2.39
Chili pepper	20	18.55 ± 1.00	92.75	5.42	19.17 ± 0.49	95.83	2.58
	10	8.61 ± 0.47	86.18	5.44	9.26 ± 0.35	92.63	3.81
	5	4.89 ± 0.14	97.95	2.93	4.81 ± 0.08	97.15	1.57

## Data Availability

The original contributions presented in this study are included in the article. Further inquiries can be directed to the corresponding authors.

## Abbreviations

The following abbreviations are used in this manuscript:

IFP	Isofenphos-methyl
OP	Organophosphorus pesticide
ic-ELISA	Indirect competitive enzyme-linked immunosorbent assay
ELISA	Enzyme-linked immunosorbent assay
mAbs	Monoclonal antibodies
MRL	Maximum residue limit
GC	Gas chromatography
GC-MS	Gas chromatography–tandem mass spectrometry
LC-MS/MS	Liquid chromatography–tandem mass spectrometry
SERS	Surface-enhanced Raman spectroscopy
SFC-MS/MS	Supercritical fluid chromatography–tandem mass spectrometry
ICA	Immunochromatographic assay
HPLC	High-performance liquid chromatography
HRMS	High-resolution mass spectrometry
NMR	Nuclear magnetic resonance
BTG	Bovine thyroglobulin
BSA	Bovine serum albumin
DMF	N, N-dimethylformamide
NHS	N-Hydroxysuccinimide
EDC	O-1-ethyl-3-(3-dimethylaminopropyl) carbodiimide
TMB	3, 3′, 5, 5′-Tetramethylbenzidin
PBS	Phosphate-buffered saline
ESP	Electrostatic potential
ALIE	Average local ionization energy
DCM	Dichloromethane
TEA	Triethylamine
THF	Tetrahydrofuran
NaH	Sodium hydride
CBS	Carbonate–bicarbonate buffer solution
OD	Absorbance
CR	Cross-reactivity
RSD	Relative standard deviation
